# Annotation of mammalian primary microRNAs

**DOI:** 10.1186/1471-2164-9-564

**Published:** 2008-11-27

**Authors:** Harpreet K Saini, Anton J Enright, Sam Griffiths-Jones

**Affiliations:** 1 Wellcome Trust Sanger Institute, Wellcome Trust Genome Campus, Hinxton Cambridge, CB10 1SA, UK; 2 Faculty of Life Sciences, University of Manchester, Michael Smith Building, Oxford Road, Manchester, M13 9PT, UK; 3 EMBL-European Bioinformatics Institute, Wellcome Trust Genome Campus, Hinxton, Cambridge, CB10 1SD, UK

## Abstract

**Background:**

MicroRNAs (miRNAs) are important regulators of gene expression and have been implicated in development, differentiation and pathogenesis. Hundreds of miRNAs have been discovered in mammalian genomes. Approximately 50% of mammalian miRNAs are expressed from introns of protein-coding genes; the primary transcript (pri-miRNA) is therefore assumed to be the host transcript. However, very little is known about the structure of pri-miRNAs expressed from intergenic regions. Here we annotate transcript boundaries of miRNAs in human, mouse and rat genomes using various transcription features. The 5' end of the pri-miRNA is predicted from transcription start sites, CpG islands and 5' CAGE tags mapped in the upstream flanking region surrounding the precursor miRNA (pre-miRNA). The 3' end of the pri-miRNA is predicted based on the mapping of polyA signals, and supported by cDNA/EST and ditags data. The predicted pri-miRNAs are also analyzed for promoter and insulator-associated regulatory regions.

**Results:**

We define sets of conserved and non-conserved human, mouse and rat pre-miRNAs using bidirectional BLAST and synteny analysis. Transcription features in their flanking regions are used to demarcate the 5' and 3' boundaries of the pri-miRNAs. The lengths and boundaries of primary transcripts are highly conserved between orthologous miRNAs. A significant fraction of pri-miRNAs have lengths between 1 and 10 kb, with very few introns. We annotate a total of 59 pri-miRNA structures, which include 82 pre-miRNAs. 36 pri-miRNAs are conserved in all 3 species. In total, 18 of the confidently annotated transcripts express more than one pre-miRNA. The upstream regions of 54% of the predicted pri-miRNAs are found to be associated with promoter and insulator regulatory sequences.

**Conclusion:**

Little is known about the primary transcripts of intergenic miRNAs. Using comparative data, we are able to identify the boundaries of a significant proportion of human, mouse and rat pri-miRNAs. We confidently predict the transcripts including a total of 77, 58 and 47 human, mouse and rat pre-miRNAs respectively. Our computational annotations provide a basis for subsequent experimental validation of predicted pri-miRNAs.

## Background

MicroRNAs (miRNAs) are short (21–23 nt), non-coding RNAs present in diverse organisms that regulate gene expression via the RNA silencing machinery. miRNAs can induce translational repression of a target transcript and/or mRNA degradation depending to some extent on the degree of complementarity between the miRNA and binding sites in the 3' untranslated regions (3'UTR) of its target [1-3]. A number of miRNAs have been implicated in the pathogenesis of human diseases, such as neurodegenerative disorders, cancer, and more recently in viral and metabolic diseases [4-11].

Previous studies have suggested that genes encoding miRNAs are surprisingly long, given the size of the processed mature final product. The miRNA biogenesis process is well-elucidated, and involves two intermediate transcript species [12-15]: The primary transcript (pri-miRNA), which can be several thousand bases long, is cleaved by the ribonuclease enzyme Drosha in the nucleus to a shorter, 70 nt stem-loop structure known as the precursor (pre-) miRNA. A subset of intronic miRNAs, known as mirtrons, bypass Drosha processing and are spliced from the intron [16-18]. The pre-miRNA is exported to the cytoplasm by the export factor Exportin 5 [19], where it is cleaved by the Dicer enzyme to form the mature miRNA [13,20]. Finally, the mature miRNA is incorporated into a ribonuclear particle (RNP), which becomes the RNA-induced gene silencing complex (RISC), capable of executing RNA-based gene silencing [21,22]. A large number of studies have been directed at understanding the processing of mature miRNAs and their target recognition. However, few studies exist pertaining to the structure of the primary miRNA transcripts [14,23-26]. Indeed, while the genomic coordinates and structures of precursor miRNA and mature miRNAs are easily obtained, there are only a handful of mammalian pri-miRNAs whose complete structures are determined experimentally [25-29]. Thus, there is a need to predict the transcript structure of pri-miRNAs and to demarcate their 5' and 3' boundaries. Such studies will help us to locate transcriptional regulatory motifs, facilitate our understanding of the regulation of miRNA expression and provide information required to make target constructs for miRNA knockouts.

Previous studies attempted to predict the transcript boundaries of pri-miRNAs based on features such as expressed sequence tags (ESTs) and transcription factor binding sites (TFBS) ([30-32]). Recently, we described a large-scale analysis of distribution of transcription features in the flanking regions of human pre-miRNAs [23]. This study showed that many transcription start sites (TSSs) and CpG islands lie within 2 kb of the precursor, but a small number appear to be 10s of kb upstream. Using other features in combination proved to be useful for predicting pri-miRNA boundaries. However, our previous study focused only on human sequences and was able to predict the putative boundaries for a limited set of pri-miRNAs. It is known that miRNAs are well conserved across a wide range of species, so it is of interest to determine whether pri-miRNAs have conserved transcript structures. Furthermore, identifying the consensus features of conserved miRNAs facilitates the prediction of transcript boundaries of a larger set of miRNAs.

We have analyzed a combination of predicted transcriptional features (TSSs, CpG islands and polyadenylation (polyA) signals) and direct evidence (ESTs, cDNAs, cap analysis of gene expression (5' CAGE) and gene identification signature (GIS) ditags) in order to predict the 5' and 3' boundaries of pri-miRNAs. We have used three closely related genomes, human, mouse and rat, to obtain sets of conserved and non-conserved pre-miRNAs using bidirectional BLAST and conserved synteny analysis. Each set is then surveyed for transcription features in their flanking regions, and transcriptional boundaries annotated. We describe here the characteristics of the predicted pri-miRNA transcripts.

## Results and discussion

### Obtaining conserved pre-miRNAs

Pre-miRNAs from the three genomes (human, mouse and rat) are divided into four groups (i) Group I: pre-miRNAs conserved in all three genomes, (ii) Group II: pre-miRNAs conserved in two of the three genomes, (iii) Group III: pre-miRNAs that are unique to one of the three genomes, but have multiple paralogous copies, and (iv) Group IV: singleton pre-miRNAs unique to one of three genomes.

#### Group I pre-miRNAs

**Figure 1:**
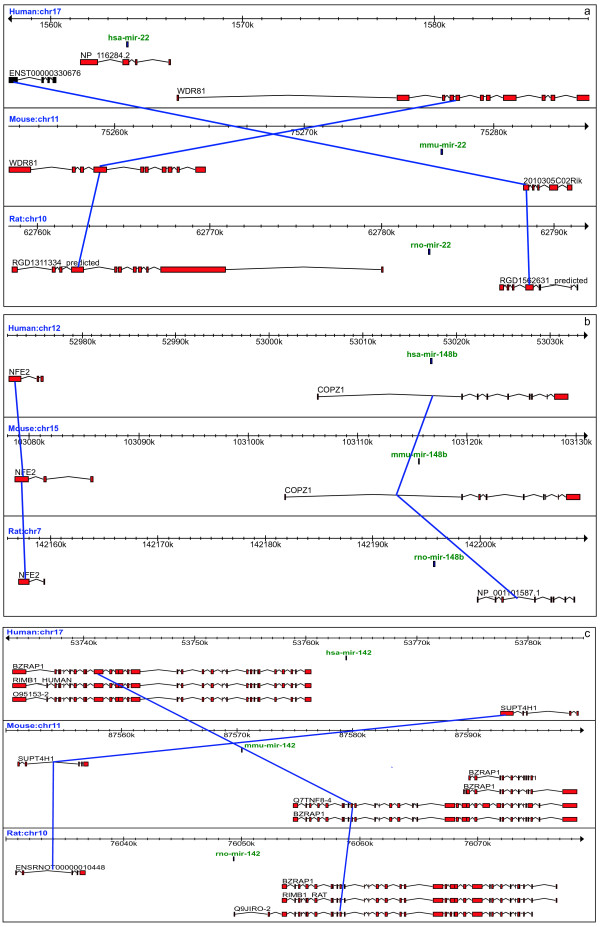
**Examples of inconsistent genomic contexts of conserved pre-miRNAs in human, mouse and rat.** Connecting lines indicate orthology. (a) Human mir-22 is located in the exon of the RefSeq gene (accession NP_116284.2), which has no annotated ortholog in mouse and rat; (b) Human and mouse miR-148b are located in the first intron of the COPZ1 transcript. The terminal 5' exon of orthologous transcript NP_001101587.1 in rat is missing, locating miR-148b upstream of NP_001101587.1; (c) Rat miR-142 is located in the first exon of the alternatively spliced transcript Q9JIR0-2. The orthologous alternative transcripts in human and mouse lacks the 5' exon, placing human and mouse miR-142 upstream of the BZRAP1 transcript.

A total of 246 annotated pre-miRNAs (miRBase release 10.0) are found to be conserved in human, mouse and rat. We looked at their genomic location to determine the fraction of miRNAs overlapping protein-coding genes and between annotated genes. We also examine whether the genomic location is similar among species. We found 114 pre-miRNAs located in intergenic sequences, 66 overlapping Ensembl genes, and, surprisingly, the remaining 66 exhibited different genomic contexts between species (categorized as "mixed" – see Table 1). A careful analysis of such inconsistencies in genomic locations in spite of conserved synteny shows that differences arise because of different host gene annotation (both presence/absence and structure) in the 3 organisms (Figure 1). For example, human mir-22 overlaps the exon of the RefSeq gene (accession NP_116284.2), which has no annotated ortholog in mouse and rat. Differences in the structure of orthologous genes (particularly 5' terminal exons, and annotation of alternative transcripts) may also cause an intronic miRNA in one organism to be annotated as intergenic in another. While these observations may reflect real gene content and structure differences, many are likely due to mis-annotation of potential miRNA host genes. For example, it is reported that the majority of vertebrate gene annotations may have missing 5' exons [33].

**Table 1:** Distribution of conserved and non-conserved pre-miRNAs in the human, mouse and rat genomes, with respect to protein-coding gene annotation.

		**Genomic Location**	
	**Intergenic**	**Intronic**	**Mixed**
		**Group I**	
**Human-Mouse-Rat**	114	66	66
		**Group II**	
**Human-Mouse**	20	28	7
**Human-Rat**	3	0	0
**Mouse-Rat**	18	4	4
		**Group III**	
**Human**	67	16	
**Mouse**	13	32	
**Rat**	1	3	
		**Group IV**	
**Human**	47	107	
**Mouse**	31	35	
**Rat**	1	4	

#### Group II pre-miRNAs

We obtained 55 pairs of human-mouse pre-miRNAs, which are not conserved in rat. Similarly, we obtained 3 pairs of human-rat conserved pre-miRNAs and 26 pairs of mouse-rat conserved pre-miRNAs (Table 1).

#### Group III pre-miRNAs

We found 237 human miRNAs with no identified orthologs in mouse and rat. Similarly, we obtained 111 and 9 miRNAs in mouse and rat respectively that lack an annotated ortholog in the other 2 species. 83 out of 237 human miRNAs group into 21 paralogous families, according to an all-against-all BLASTN search (E-value ≤ 1 × 10^-5^) (Table 2). The largest set of paralogs comprises 39 members clustered on chromosome 19 (family 17). We observe that 12 paralogous families with one or more miRNAs have previously defined orthologs in mouse and/or rat, and are therefore included in groups I and II above. For instance, the human genome has 3 paralogous mir-199 genes. hsa-mir-199a-2 has defined orthologs in both mouse and rat, and hsa-mir-199b is orthologous to mmu-mir-199b. Such paralogous families are shown (Table 2). 45 mouse and 4 rat miRNAs grouped into 12 and 2 paralogous families respectively (Tables 3 and 4). The largest paralogous family in mouse represents 23 miRNAs, all located on chromosome 2 (family 7).

**Table 2:** Paralogous families of human pre-miRNAs.

Family	Members
1	mir-105-1 mir-105-2^†m^
2	mir-147 mir-147b^††^
3	mir-153-1 mir-153-2^††^
4	mir-199a-1 mir-199a-2^†† ^mir-199b^†m^
5	mir-220a mir-220b
6	mir-329-2 mir-329-1^††^
7	mir-374a mir-374b^††^
8	mir-376a-2 mir-376a-1^†† ^mir-376b^†† ^mir-376c^††^
9	mir-422a mir-378^††^
10	mir-450a-2 mir-450a-1^†† ^mir-450b^†m^
11	mir-487a mir-487b^†† ^mir-539^†† ^mir-154^††^
12	mir-500 mir-501^†† ^mir-502^††^
13	mir-509-1 mir-509-2 mir-509-3 mir-514-1 mir-514-2 mir-514-3 mir-510
14	mir-511-1 mir-511-2^†m^
15	mir-512-1 mir-512-2
16	mir-513a-1 mir-513a-2 mir-513c mir-513b
17	mir-520e mir-520c mir-524 mir-515-2 mir-515-1 mir-525 mir-519b mir-519a-2 mir-520f mir-516b-2 mir-519d mir-516a-2 mir-526a-1 mir-517c mir-518e mir-521-2 mir-516a-1 mir-516b-1 mir-518c mir-527 mir-520g mir-526b mir-520b mir-517a mir-519e mir-526a-2 mir-518f mir-522 mir-517b mir-518a-2 mir-519c mir-523 mir-520d mir-521-1 mir-520h mir-518d mir-520a mir-518b mir-518a
18	mir-550-1 mir-550-2
19	mir-570 mir-548c mir-548d-2 mir-548a-3 mir-548b mir-548d-1 mir-603 mir-548a-1 mir-548a-2
20	mir-891a mir-891b
21	mir-941-1 mir-941-2 mir-941-3 mir-941-1

^†† ^orthologs in mouse and rat. ^†m ^orthologs in mouse only.

**Table 3:** Paralogous families of mouse pre-miRNAs.

Family	Members
1	mir-135a-1 mir-135a-2^†† ^mir-135b^††^
2	mir-199a-1 mir-199b ^†h ^mir-199a-2^††^
3	mir-291b mir-291a^†r^
4	mir-344-1 mir-344-2^†h^
5	mir-450a-2 mir-450a-1^††^
6	mir-465a mir-465b-2 mir-465b-1 mir-465c-1 mir-465c-2^†h^
7	mir-466a mir-466e mir-466c mir-466b-1 mir-466b-3 mir-466b-2 mir-466d mir-466h mir-466g mir-669a-1 mir-669a-2 mir-669a-3 mir-466f-1 mir-669c mir-669b mir-297b mir-297c mir-466f-3 mir-466f-2 mir-297a-4 mir-297a-3 mir-297a-5 mir-297a-2
8	mir-467a mir-467b mir-467c mir-467d mir-467e
9	mir-680-1 mir-680-2 mir-680-3
10	mir-684-1 mir-684-2
11	mir-692-1 mir-692-2
12	mir-883b mir-883a^†r^

^†† ^orthologs in mouse and rat. ^†h ^orthologs in human only. ^†r ^orthologs in rat only.

**Table 4:** Paralogous families of rat pre-miRNAs.

Family	Members
1	mir-466b-1 mir-466b-2
2	mir-664-1 mir-664-2

#### Group IV pre-miRNAs

There are 154 singleton human miRNAs with no defined homologs. We also find 66 mouse and 5 rat singleton miRNAs (Table 1). These may represent species-specific miRNAs. It is also likely that with ongoing miRNA discovery and the addition of new sequences to miRBase, some singleton miRNAs may find relationships to new miRNAs.

### Annotation of pri-miRNAs

We analyzed different transcriptional features in the flanking regions of miRNAs, in order to predict the putative boundaries of their primary transcripts. It is widely assumed that intronic miRNAs are generally transcribed coincidentally with their host genes. The pri-miRNA in these cases is therefore the host protein-coding transcript. We therefore focus on predicting primary transcripts of intergenic miRNAs (that is between protein-coding gene annotations). The 5' ends of pri-miRNAs are annotated based on the mappings of predicted TSS, CpG islands and 5' CAGE tags to the upstream flanking regions. Similarly, the 3' end is demarcated based on predicted polyA signals and 3' ditags in the downstream flanking region. Further, these predictions are supported by transcriptional evidence, either from cDNA or ESTs. Highly confident annotations are obtained for 59 pri-miRNAs, with 36 pri-miRNAs conserved in all 3 species (Group I), 4 pri-miRNAs conserved in only two species (Group II), 15 human unique pri-miRNAs and 4 mouse unique pri-miRNAs. The predicted transcript structures are also analyzed for functional regulatory regions such as promoter-associated regulatory sequences and CTCF-enriched insulator sites surrounding the putative 5' ends.

#### Group I Pri-miRNAs

**Figure 2:**
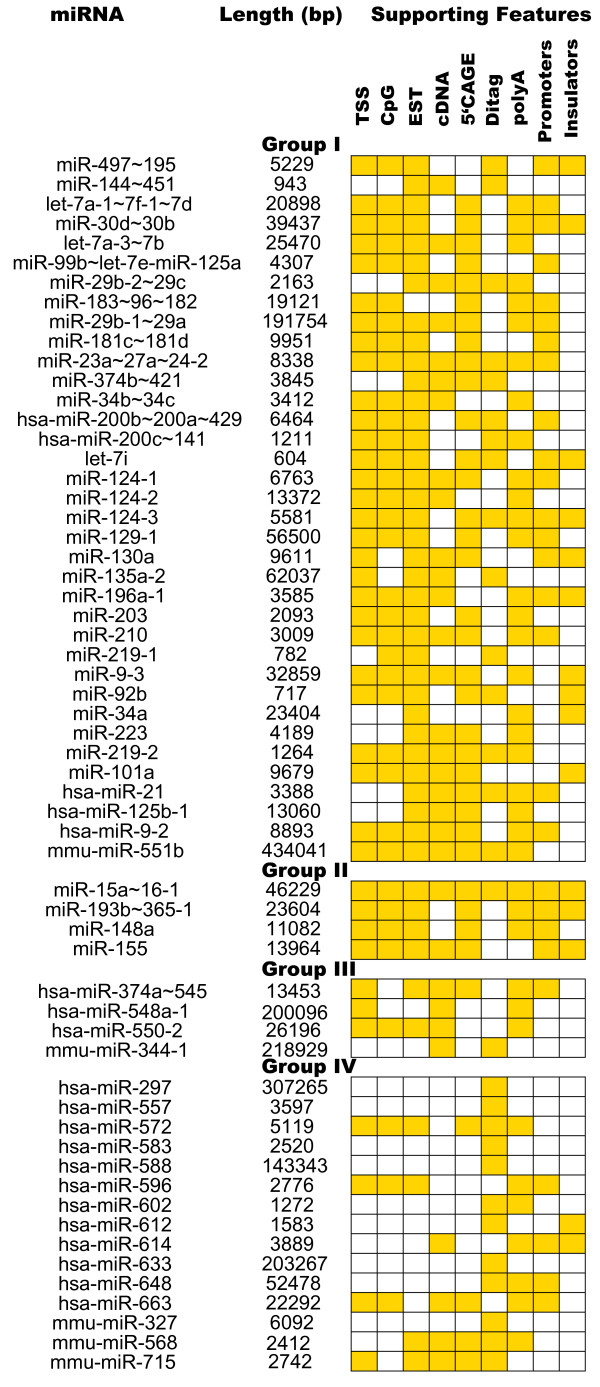
**Predicted pri-miRNAs, their lengths, and features that support the pri-miRNA prediction.** The presence and absence of a feature is shown by colored and open boxes respectively.

We annotate 36 predicted pri-miRNAs conserved in human, mouse and rat, expressing mature products from 56 pre-miRNAs. Among them, 15 pri-miRNAs are polycistronic and the remaining 21 each contain a single miRNA hairpin. 7 of the predicted polycistronic transcripts are found to be completely overlapped by ESTs/cDNAs (Table 5). For instance, the cluster mir-29b-2~29c on chromosome 1 in mouse is completely overlapped by a cDNA (accession AK081202) spanning approximately 211 bp upstream from the 5' end of the cluster to 1,274 bp downstream from the 3' end of the cluster. Predicted lengths of pri-miRNAs and features supporting their annotation are shown (Figure 2). The predicted genomic coordinates of pri-miRNAs are provided in Additional file 1. Here, we describe in detail the annotation of three pri-miRNAs.

**Table 5:** Polycistronic clusters of intergenic miRNAs with complete EST/cDNA coverage.

**Cluster**	**Mapped ESTs**	**Mapped cDNAs**
mir-497~195	Human: CR737132, DB266639, DA2895925, BI752321, AA631714	Human: AK098506.1
	Rat: CV105515	
mir-144-451	Human: R28106	Mouse: AK158085.1
	Rat: AW919398, BF2869095, AI008234	
mir-99b~let-7e~mir-125a	Human: DB340912	Human: AK125996
mir-143~145	Human: BM702257	
mir-181a-1~181b-1	Human: DA528985, BX355821	
	Mouse: BE332980, CA874578	
mir-29b-2~29c	Human: BF089238	Mouse: AK081202, BC058715
mir-298~296	Human: W37080	
mir-183~96~182	Human: CV424506	
mir-181c~181d	Human: AI801869, CB961518, CB991710, BU729805, CB996698, BM702754	
	Mouse: CJ191375	
mir-100~let-7a-2	Human: DA545600, DA579531, DA474693, DA558986, DA600978	Human: AK091713
	Mouse: BB657503, BM936455	
	Rat: BF412891, BF412890, BF412889, BF412895	Mouse: AK084170
mir-374b~421	Human: DA706043, DA721080	Human: AK125301
	Rat: BF559199, BI274699	Mouse: BC027389, AK035525, BC076616, AK085125
mir-34b~34c		Human: BC021736
mir-15a-16-1	Human: BG612167, BU932403, BG613187, BG500819	Human: BC022349, BC022282, BC070292, BC026275, BC055417, AF264787
	Mouse: AI789372, BY718835	Mouse: AK134888, AF380423, AF380425, AK080165
mir-193b~365-1	Human: BX108536	
hsa-mir-200c~141	Human: AI969882, AI695443, AA863395, BM855863.1, AA863389	
mir-374a~545	Human: DA685273, AL698517, DA246751, DA755860, CF994086, DA932670, DA182706	Human: AK057701

##### let-7i

**Figure 3:**
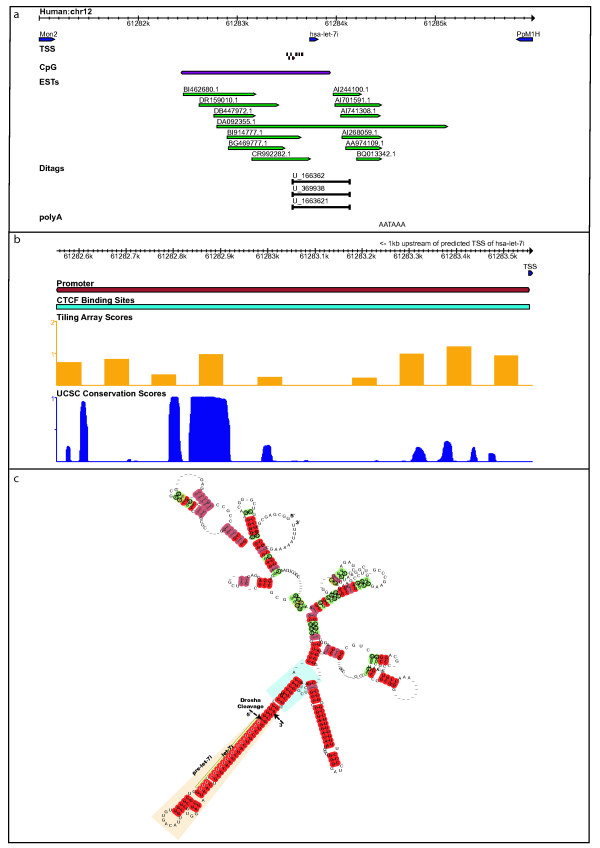
**Annotation of pri-let-7i.** (a) Transcription features mapped in the flanking regions surrounding let-7i in human; (b) Predicted promoter and insulator associated regulatory regions upstream of pri-let-7i and the corresponding tiling array and UCSC conservation scores; (c) RNAalifold-predicted secondary structure of pri-let-7i. The pairs conserved in human, mouse and rat are marked in red. The hairpin precursor and the mature sequence are boxed. The Drosha cleavage sites are marked by a dashed black arrow. The stem segments and basal single-stranded regions immediately flanking the pre-miRNA are highlighted in blue.

The miRNA let-7i is conserved in all 3 species, located on chromosomes 12, 10 and 7 in human, mouse and rat respectively. The flanking upstream and downstream orthologous genes are monensin sensitive gene (Mon2) and protein phosphatase 1H (PpM1H). Six transcription feature types (TSS, CpG, 5'CAGE, ESTs, Ditags and polyA) support the structure of pri-let-7i. In human there are 7 closely situated TSS predictions, on average 188 bp upstream of 5' end of the precursor (Figure 3a). We identify CpG islands overlapping the predicted TSS, strongly supporting the 5' end annotation. 7 ESTs overlap with 5' ends close to the predicted TSS and CpG islands. Among them, EST 'DA092355' completely overlaps hsa-let-7i. In mouse and rat, there are more than 10 tightly clustered TSS predictions, at 211 bp and 297 bp upstream of the precursor in mouse and rat respectively (data not shown). The 5' end is also supported by predicted CpG islands and FANTOM 5'CAGE tags in mouse at ~200 bp upstream of the start of the precursor. In rat, a CpG island at ~430 bp upstream of rno-let-7i overlaps with the predicted TSS. These upstream features strongly suggest that the 5' end of pri-let-7i in mouse and rat is within ~300 bp of the pre-miRNA. Moreover, the closely clustered TSS predictions suggest a "broad" promoter, with each TSS representing an independent form of the pri-miRNA [34]. In human, three 5' ditags (U_166362, U_1663621 and U_369938) overlap with the predicted TSS/CpG as shown (Figure 3a). The associated 3' tags are located at ~300 bp downstream of let-7i. We identify a polyA signal 'AATAAA' at ~612 bp from the 3' end of hsa-let-7i, which overlaps with the 3' ends of 6 ESTs (accessions: AI244100, AI701591, AI741308, AI268059, AA974109 and BQ013342). Hence, it can be concluded that the 3' end of pri-let-7i in human is situated within ~600 bp of the 3' end of the precursor. In mouse, 2 polyA signals, 'AATAAA' and 'TATAAA ' predict the 3' end of the pri-miRNA to be ~530 bp downstream of the precursor, supported by 7 ESTs and a cDNA (accession AK052706) within 10 bp of the predicted polyA signals. In rat, 6 ESTs and a cDNA support a strongly conserved 3' end. Based on these analyses, we conclude that the distribution of transcription features around let-7i is similar in human, mouse and rat and their pri-miRNAs are strongly conserved in lengths and boundaries (603, 604, and 604 bps in human, mouse and rat respectively).

Further, we analyzed the regulatory features such as promoters and insulator sequences in the upstream region of the predicted human pri-let-7i. Insulators are sequences located between enhancers and promoters of adjacent genes and prevent an enhancer from inappropriately binding to and activating the promoter of a neighbouring gene. In vertebrates, insulator function requires association with CCCTC factor (CTCF) binding sites. The normalized chromatin immunoprecipitation genome-tiling (ChIP-chip) array scores for CTCF binding sites and the sequence conservation of the regulatory features in the upstream regions of mouse and rat pri-let-7i are shown (Figure 3b). We identify promoter sequences and CTCF binding sites spanning a region from 61,282.5 kb to 61,283.5 kb, ~1 kb upstream of the predicted 5' end of pri-let-7i. The corresponding regions in mouse and rat show a strong conservation in relative position, suggesting the putative promoter regions. Analyzing these regions using the UCSC conserved transcription factors track allowed us to identify two conserved transcription factors binding sites: activating transcription factor 6 (ATF6) and upstream transcription factor 1 (USF1) located at ~61,282.8 kb, which may be important for let-7i expression. However, delineating the transcription factors that bind in the promoter region requires further analysis and experimental validations.

We predicted the consensus secondary structure of pri-let-7i based on the sequence alignments of human, mouse and rat sequences, using RNAalifold (Figure 3c) [35]. The conserved pair residues are marked in red. It can be seen that the stem segments immediately flanking the pre-miRNA are conserved (blue box). Previous studies have also shown that the sequences flanking the miRNA hairpin are important for miRNA biogenesis [16,36]. In particular, the stem extension located immediately adjacent to the pre-miRNA hairpin and the single-stranded basal segments at the ends are required for efficient processing by Drosha [37,38].

##### miR-23a~27a~24-2

**Figure 4:**
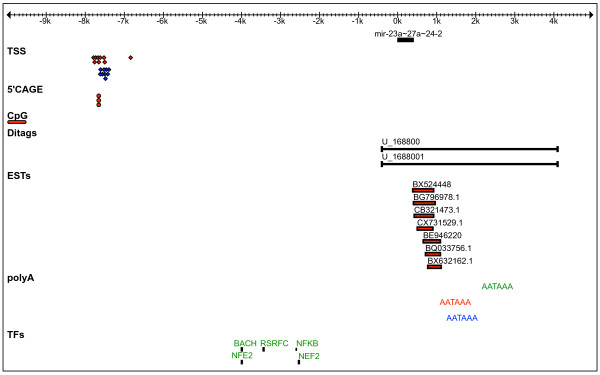
**Consensus display of transcription features mapped in the flanking regions surrounding the cluster mir-23a~27a~24-2 in human, mouse and rat.** The distances are relative to the 5' end of the miR-23a precursor. The human, mouse and rat mir-23a~27a~24-2 features are shown in green, red and blue respectively.

The order, orientation and relative spacing of the cluster miR-23a~27a~24-2 and its neighbouring genes, nanos homolog 3 (Nanos3) and zinc finger SWIM-type containing 4 (Zswim4), are conserved in all three species. The mapping of transcription features relative to the start and end of the precursor is shown (Figure 4). Eponine predicts 9 TSSs at an average distance of 7,560 bp and 7,504 bp upstream of the start of the miR-23a in mouse and rat respectively, but none in human. The predicted TSS in mouse is supported by 3 FANTOM 5'CAGE tags at 7,651 bp from the start of mmu-miR-23a. CpG islands are predicted in close proximity to the TSS and 5'CAGE mapping. Taken together, these data suggest that the 5' end of the pri-miRNA miR-23a~27a~24-2 is ~7500 bp upstream of miR-23a. Although there is no evident feature supporting the 5' end of the human pri-miRNA, we identify two ditags, U_168800 and U_1688001, with their 5' tags located at 394 bp upstream of hsa-miR-23a. No EST/cDNA data supports the 5' end of the pri-miRNA in any of the species. For all three species, we identify a polyA signal 'AATAAA' at 1,751 bp, 669 bp and 845 bp downstream of miR-24-2 in human, mouse and rat respectively. The 3' end of the human pri-miRNA cluster at 1751 bp is also in agreement with previous studies [25], although we note that the 5' boundary differs from that determined experimentally. In mouse, overlapping ESTs (accessions: BX632162, BG796978, CX731529, CB321473, BE946220, BX524448, BQ033756) support a 3' end located ~619 bp downstream of miR-24-2, in close proximity to the predicted polyA signals (Figure 4). Based on these, we predict the genomic coordinates of the polycistronic primary transcript of miR-23a~27a~24-2 in human, mouse and rat (see Additional file 1), to be 6,425 bp, 9,320 bp and 9,270 bp in length respectively. For the human miRNA cluster, we also obtained promoter-associated regions around 2 kb upstream of the pri-miRNA. The predicted transcription factors (TFs) binding sites are shown (Figure 4) and the corresponding regions in mouse and rat are found to be conserved.

##### miR-124-1

**Figure 5:**
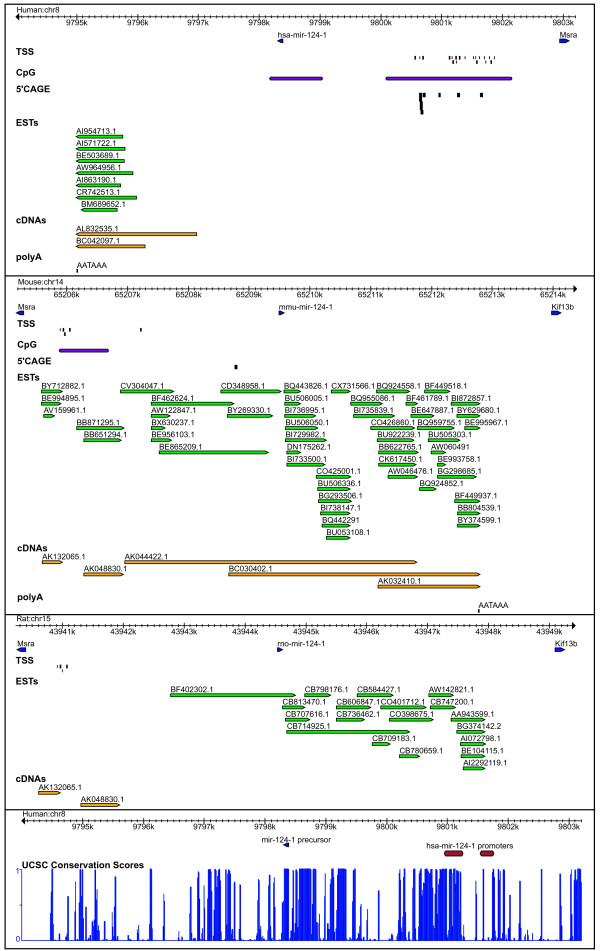
**Transcription features mapped in the flanking regions surrounding mir-124-1 in human, mouse and rat.** The lower panel shows the promoter region and UCSC conservation scores along the length of predicted pri-hsa-mir-124-1.

The brain-specific miRNA, miR-124-1 is an intergenic miRNA conserved in all the 3 species, flanked by genes methionine sulfoxide reductase A (MSRA) and Kinesin family member 13B (Kif13b), although the human syntenic region lacks the Kif13b gene annotation (Figure 5). mir-124-1 has the highest number of TSS predictions of all miRNAs in this study, the majority falling within 3,500 bp upstream of mir-124-1 in all 3 species. The TSS predictions are also supported by CpG islands (3,766 bp and 3,624 bp upstream of miR-124-1 in human and mouse respectively) and 5' CAGE tags. Moreover, in human, miR-124-1 is embedded in the CpG island, suggesting the intriguing possibility that its expression may be regulated by an epigenetic mechanism involving methylation of the CpG island. The human miR-124-1 has 15 tightly clustered 5'CAGE tags within 90 bp of the predicted TSS, strongly supporting the 5' end of pri-miRNA of hsa-mir-124-1 (Figure 5). In mouse, the predicted TSS/CpG is further supported by 3 overlapped ESTs (accessions: BY712882.1, BE994895.1 and AV159961.1) and 1 cDNA (accession AK132065.1), which are located 3900 bp upstream of mmu-miR-124-1 (Figure 5). The polyA signal 'AATAAA' in the downstream region is located at 3,337 bp and 3,196 bp from the 3' end of pre-miRNA in human and mouse respectively. The polyA signal prediction is corroborated in human, mouse and rat by multiple ESTs with 3' ends aligned between 3,200 and 3,500 bp downstream from the 3' end of miR-124-1. The predicted genomic coordinates of pri-mir-124-1 are given in Additional file 1. We also identify promoter-associated regulatory features and DNase1 hypersensitive sites in the region 9800.9–9801.8 kb upstream of human mir-124a-1, overlapping with predicted TSS/CpG, and the corresponding region is found to be conserved in mouse and rat (Figure 5). The promoter region is found to contain predicted binding sites for the transcription factors HUA enhancer (HEN), X-box binding protein (XBP), homeobox protein (HOX13) and NF-E2-related factor 2 (NRF2).

#### Group II pri-miRNAs

We annotate the boundaries of four pri-miRNAs conserved in 2 of the 3 genomes. Among them are two polycistronic transcripts (miR-15a~16-1 and miR-193b~365-1), and two expressing single miRNAs (miR-148a and miR-155). Figure 2 shows the predicted length of the pri-miRNAs and the features supporting them. The predicted genomic coordinates of pri-miRNAs are provided in Additional file 1. Here, we describe in detail the annotation of the pri-miRNA containing miR-15a and miR-16-1.

##### miR-15a~16-1

**Figure 6:**
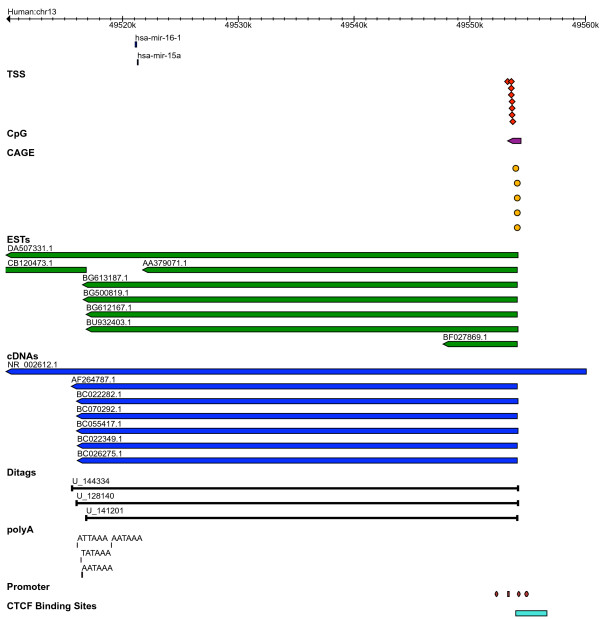
**Transcription features mapped in the flanking regions surrounding the cluster mir-15a~16-1 in human.** The lower panel shows the promoter, CTCF binding regions upstream of predicted TSS and the corresponding UCSC conservation scores.

The structure of a polycistronic transcript expressing miR-15a and miR-16-1 is strongly supported by all seven types of transcriptional features. The cluster is located between the genes Dleu (deleted in lymphocytic leukemia) and potassium channel regulator (KCNRG) on chromosome 13 and 14 in human and mouse respectively. The features mapped to the flanking regions surrounding the hsa-mir-15a~16-1 are shown (Figure 6). In human, we predict 8 TSSs with an average distance of 32,242 bp upstream of miR-15a. The predicted TSSs are also overlapped by CpG islands located at 31,945 bp. A similar distribution of TSS and CpG islands is observed in mouse at a larger distance (~57,0101 bp). Five 5' CAGE tags and three 5' ditags (U_144334, U_1281401 and U_141201) are located within 300 bp of the predicted TSS in both species and the 5' ends of multiple ESTs/cDNAs overlap the predicted TSS/CpG/5'CAGE (within 55 bp, Figure 6). In mouse, 3 ESTs and 4 cDNAs overlap the entire miRNA cluster. Taking all these features together, we annotate the 5' end of the human pri-miRNA miR-15a~16-1 at ~33 kb upstream of miR-15a.

PolyA signals 'AATAAA', 'ATTAAA' and 'TATAAA' are predicted at an average distance of 4,695 bp and 4,595 bp from the 3' end of miR-16-1 in human and mouse respectively. The 3' end is also supported by ditags in human (U_144334, U_1281401 and U_141201), 4,208 bp from the 3' end of miR-16-1, and by ESTs and cDNAs in both human and mouse (Figure 6). We conclude that the 3' boundaries of human and mouse pri-miRNAs are similar, but that the length of the 5' upstream transcript is significantly different. The respective genomic coordinates and predicted lengths of pri-miRNAs are shown (Figure 2 and table S1). We identify promoter and CTCF binding sites (average tiling array score = 1.36) ~150 bp upstream of the predicted TSS in human, with the corresponding region conserved in mouse (Figure 6).

These data agree with previous annotation by the VEGA project of non-protein-coding transcripts (accessions: OTTHUMT00000044959 and OTTHUMT00000044961) expressing miR-15a and miR-16-1 in human, called DLEU2 [39]. This region has been shown to be deleted or down-regulated in chronic lymphocytic leukaemia cases [40].

#### Species-specific (Group III and IV) pri-miRNAs

**Figure 7:**
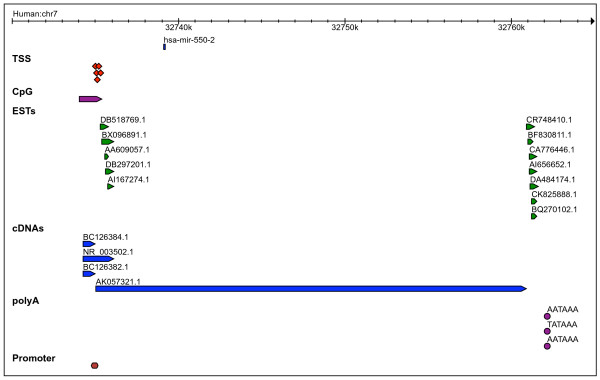
Transcription features mapped in the flanking regions surrounding hsa-mir-550-2.

**Figure 8:**
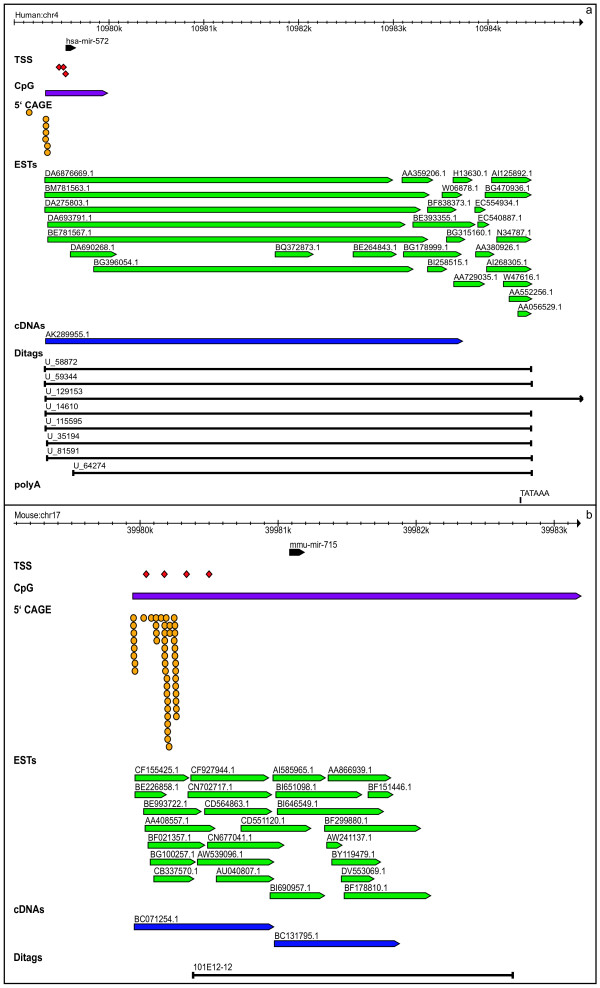
Graphical display of transcription features mapped in the flanking regions surrounding miRNAs of Group IV; (a) hsa-mir-572 and (b) mmu-mir-715.

We are able to confidently predict the pri-miRNAs of four species-specific miRNAs with paralogs (group III), three in human and one in mouse. The cluster hsa-miR-374a~545 is a polycistronic transcript, as indicated by the complete overlap of 7 ESTs and 1 cDNA (Table 5). Additionally, we confidently annotate the pri-miRNAs of fifteen singleton miRNAs, comprising 12 human miRNAs and 3 mouse miRNAs. Figures 7 and 8 show examples of the pri-miRNA structure of species-specific miRNAs. Genomic coordinates are provided in Additional file 1.

### Characteristics of Predicted Pri-miRNAs

By analyzing the transcriptional features mapped in the upstream and downstream flanking regions surrounding the precursor miRNAs, we are able to characterize the 5' and 3' ends and lengths of their primary transcripts. Several observations can be made from these analyses.

Mapping of 5'CAGE tags and prediction of polyA signals in the flanking sequences of precursor miRNAs in human, mouse and rat clearly indicate that the pri-miRNAs are both 5' capped and polyadenylated. This provides strong evidence that the major fraction of mammalian miRNAs is transcribed by RNA polymerase II (pol II). The distribution of pol II TSS predictions also supports this assumption. Previous studies have also reported that the pol II is the major polymerase of human miRNA transcription [25,26]. However, a small number of miRNAs lying within Alu repeats have been reported to be transcribed by pol III [41].

**Figure 9:**
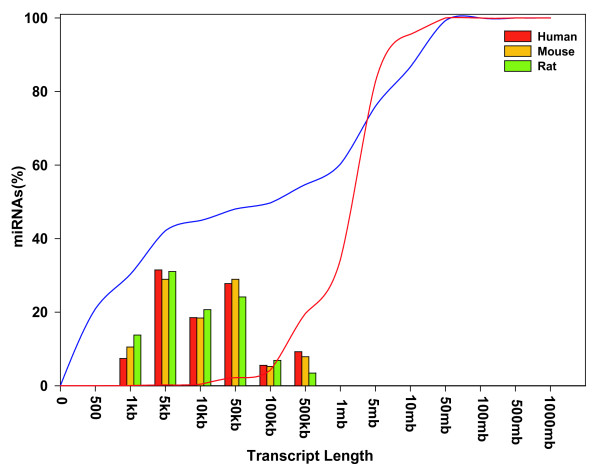
**Distribution of lengths of predicted pri-miRNAs of human, mouse and rat.** The blue curve shows the fraction of human miRNAs clustered as a function of inter-miRNA distances. The red curve shows the distribution of inter-sequence distances of an equal number of randomly generated genomic positions.

We find that 18 of 59 (31%) confidently annotated pri-miRNAs contain more than one precursor miRNA, clearly showing that clustered miRNAs are often transcribed polycistronically. 15 of the polycistrons are conserved between human, mouse and rat. Polycistronic miRNAs are highly likely to be expressed at the same time and location, and therefore to be functionally related. The 41 precursor miRNAs in 18 transcripts are organised as 13 pairs and 5 triplets. We also found 4 additional polycistronic miRNA clusters supported by EST/cDNA data, though we were not able to confidently predict the 5' and 3' ends of their primary transcripts. Interestingly, the length distributions of polycistronic pri-miRNAs and those expressing a single miRNA are very similar – 9 of 18 (50%) of the predicted polycistronic pri-miRNAs and 21 of 41 (51%) of the single miRNAs have lengths between 1 and 10 kb. Figure 9 shows the length distributions of the predicted primary transcripts of human, mouse and rat miRNAs with mean lengths of 3903, 3983 and 4020 bp respectively. The graph also clearly shows that miRNA genes are more clustered than expected by chance. Indeed, the inflection point in the distribution of inter-miRNA distances allows us to predict that around 50% of human miRNAs are polycistronically transcribed in primary transcripts up to 10s of kb long. This is consistent with previous observations [42,43]. The mean length of a protein-coding pre-mRNA in human is around 50 kb, while, surprisingly, the mean length of a pre-mRNA that contains intronic miRNAs is over 150 kb. Non-protein-coding pri-miRNA transcripts are therefore significantly shorter than protein-coding transcripts in general, and particularly those that also express miRNAs. We note that we are able to annotate very few intergenic pri-miRNAs with lengths greater than 100 kb. We cannot rule out the possibility that this reflects an annotation bias, due to difficulties in viewing and collating transcriptional feature evidence over larger distances.

We have examined the exon-intron organization of predicted pri-miRNAs based on EST/cDNA alignments. ESTs or cDNAs spanning the entire pre-miRNA reveal that pri-miRNAs have conventional exon-intron structures, although they appear to contain fewer introns than protein-coding messages. 44% (26/59) of our annotated pri-miRNAs have good EST/cDNA alignments across the entire transcript. 92% of these have fewer than four introns (mean number of introns per transcript = 0.74). 6 pri-miRNAs are intronless. For example, the cluster mmu-mir-144~451 is overlapped by a full length cDNA 'AK158085.1', whose 5'/3' ends coincides with ditags. The set of unspliced transcripts also include pri-mir-21, which was previously shown experimentally to be intronless [26]. About 50% of the predicted pri-miRNAs have only one intron. For example pri-mir-196a-1 has one full-length cDNA with its 5'/3' ends coinciding exactly with predicted TSS, polyA and ditags.

**Figure 10:**
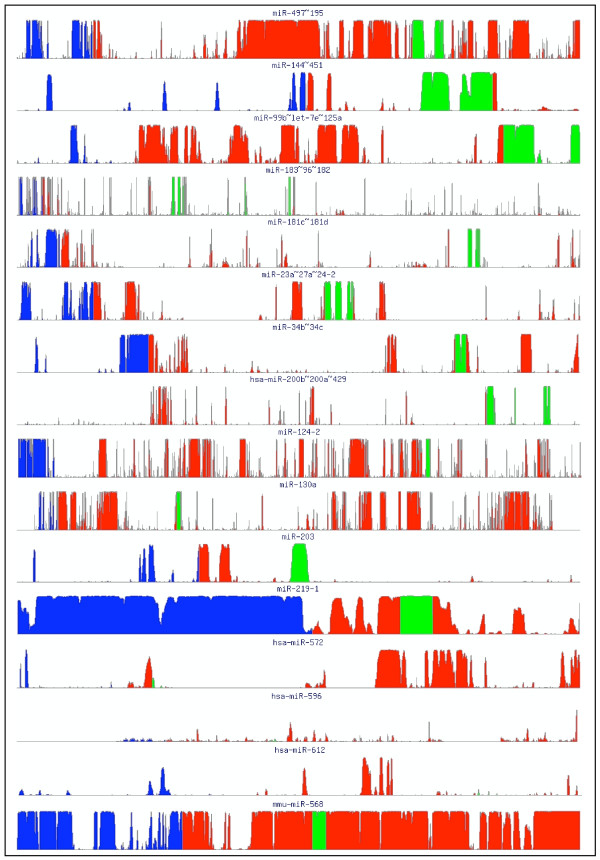
**Conservation profiles of predicted pri-miRNAs.** The predicted pri-miRNA is shown in red, the hairpin precursor sequence in green, and the 1 kb region upstream of the predicted 5' end of pri-miRNA in blue.

Finally, we analyzed the sequence conservation along the whole length of predicted pri-miRNAs, and 1 kb upstream of their putative 5' ends – examples are shown in Figure 10. Conserved sequences within the pri-miRNA may indicate regions important for miRNA biogenesis, while upstream conservation may inform on regulatory signals. As expected, the conservation is highest in the precursor sequence (shown in green) and in the segments immediately flanking the precursor. We also observe that the precursor sequences of Group IV miRNAs are poorly conserved in whole genome alignments. For example, hsa-mir-572, hsa-mir-596 and hsa-mir-612 have low conservation values as compared to the flanking sequences (Figure 10), suggesting that these miRNAs really are absent from rodent genomes. The precursor of mmu-miR-568 is very well conserved, suggesting that it is likely present in the other genomes, but not yet annotated. We also note that outside of the precursor, the most conserved regions tend to be at the ends of the predicted pri-miRNAs. A few pri-miRNAs exhibit conservation along the entire length of the pri-miRNA (for example mir-497~195, mir-99b~let-7c~mir-125a, mir-124-2, mir-130a and mmu-mir-568) (Figure 10). Some of these conserved flanking sequences may correspond to regulatory sequences, alternative transcripts or antisense transcripts. For instance, the high conservation of flanking sequence downstream of mir-497~195 can be attributed to the presence of an antisense transcript 'c17orf49'. The 1 kb region upstream of the pri-miRNA (shown in blue, Figure 10) is highly conserved in most of the pri-miRNAs, except the hsa-mir-200b~200a~429 cluster. The strong conserved blue peaks (Figure 10) upstream of pri-miRNAs may represent the putative promoter regions.

## Conclusion

Previously, very little data has been presented regarding the primary transcript structures of miRNAs. We have systematically annotated the primary transcripts of human, mouse and rat intergenic miRNAs using various transcription-related features. The 5' end of the primary transcript is predicted based on mapped TSS, CpG and 5' CAGE tags in the upstream region. The 3' end is predicted based on the mapping of polyA signals, and supported by multiple ESTs/cDNA mappings. In addition, the complete transcript structure is also supported by mapped ends of ditags. Using conservation and synteny, we are able to identify the boundaries of a significant proportion of mammalian miRNAs. We show that the transcription features in the flanking regions around conserved miRNAs have similar distribution and exhibit similar transcript structure in the three genomes. The results also indicate that a significant fraction of pri-miRNAs have lengths between 1 and 10 kb. Previous experimental studies of pri-miRNAs have also identified transcript lengths of 1–4 kb [25,26,29]. However, we also identify a small number of pri-miRNA candidates with exceptional length – up to 100s of kb. While pri-miRNAs are significantly shorter on average than protein-coding messages (including those with intronic miRNAs), the disparity between the length of the transcribed sequence and the final functional product is startling. It remains to be seen whether long non-protein-coding pri-miRNAs have function in addition to that of the miRNA itself.

## Methods

### Obtaining Pre-miRNAs sequences

The sequences and genomic coordinates of human, mouse and rat pre-miRNAs were obtained from miRBase::Sequences (version 10.0) [45]. The human, mouse and rat genome annotations were obtained from Ensembl release 48 [46]. miRNAs located outside of Ensembl transcripts were classified as "intergenic", while those overlapping annotated transcripts were classified as "intronic".

### Obtaining conserved miRNAs

We identified a set of conserved pre-miRNAs between human, mouse and rat. Reciprocal-best BLAST hits highlighted miRNA pairs that are best matched to each other. Each miRNA pair was subjected to synteny analysis, using Ensembl Compara [41]. Pairs were retained for subsequent analysis if the neighboring genes of the pre-miRNA in one species had one-to-one matches to the neighboring genes of orthologous pre-miRNA in the other species. Pre-miRNAs with no reciprocal hits are further classified as paralogs if they have homologs in the same genome, but no homologs in the other two genomes using all-against-all BLAST.

### Obtaining flanking regions

The upstream and downstream flanking sequences around human, mouse and rat pre-miRNAs were obtained from Ensembl using the Perl API (release 48), representing genome assemblies NCBI 36, NCBI m37 and RGSC 3.4 respectively. For intergenic miRNAs, we truncated the flanking region if it overlapped with any neighboring Ensembl-annotated transcript.

### Transcriptional Features

We analyzed seven different transcriptional features: transcription start sites (TSSs), CpG islands, ESTs, cDNAs, polyA signals, 5'CAGE and GIS-PET as described previously [23]. CAGE tags are 20- or 21-nt sequence tags that are derived from the mRNA sequenced in the proximity of the cap site and their mapping to unique genomic sequences identifies TSSs [47]. Ditags are 5' and 3' signatures of a full-length transcript and thus are useful in defining the transcript boundaries [48]. Additionally, regulatory features such as promoters and insulators were obtained from the Ensembl Functional Genomics database (Release 48), which includes experimental data from experiments such as DNaseI hypersensitivity sites and CTCF binding sites [46,49,50]. The conserved transcription factor binding sites in promoter regions are obtained from UCSC genome browser [51]. CTCF binding sites in the human genome are obtained from ChIP-chip experiments [52].

### Availability

The pri-miRNA annotations are available to the public as DAS sources [44] for viewing in the Ensembl genome browser or other DAS clients (,  and ). The feature sets used to annotate pri-miRNAs here are also available through the Genomics section of the miRBase database .

## Authors' contributions

HKS, AJE and SGJ conceived the project; HKS collected and analyzed the data, and drafted the manuscript; AJE and SGJ supervised the project and finalized the manuscript. All authors read and approved the final manuscript.

## Supplementary Material

Additional file 1 **Predicted primary transcripts of human, mouse and rat miRNAs.** Genomic coordinates, length and supporting evidence for predicted primary transcripts of human, mouse and rat miRNAs. Click here for file
