# Supplementary material for: Annotation of mammalian primary microRNAs
Source: BMC Genomics. 2008 Nov 27;9:564. doi: 10.1186/1471-2164-9-564 (PMC2632650; doi:10.1186/1471-2164-9-564)
Supplement: Additional file 1 — Predicted primary transcripts of human, mouse and rat miRNAs. Genomic coordinates, length and supporting evidence for predicted primary transcripts of human, mouse and rat miRNAs. [file 1471-2164-9-564-S1.doc]

**Additional File 1**

Genomic coordinates, lengths and supporting evidence for predicted primary transcripts of human, mouse and rat miRNAs.

Genome assembly versions are from Ensembl release 48.

| **Ortholog mirNA/cluster** | | **Genomic coordinates of pre-mirNA/cluster**  ***(Species: Chromosome: Coordinates [Strand])*** | **Predicted 5’ end of pri-mirNA** | **Predicted 3’ end of pri-mirNA** | **Length of the primary transcript** | **Supporting Evidences** | |
| --- | --- | --- | --- | --- | --- | --- | --- |
| **Group I pri-miRNAs** | | | | | | | |
| **mir-497~195** | | Human: 17: 6861658-6862065 [-1]  Mouse: 11: 70048219-70048637 [1]  Rat: 10: 57073846-57074256 [1] | 6866265  70044089  57069716 | 6859865  70048737  57074356 | 6400  4648  4640 | TSS, CpG, ESTs | |
| **mir-144~451** | | Human: 17: 24212513-24212762 [-1]  Mouse: 11: 77886507-77886743 [1]  Rat: 10: 64129346-64129589 [1] | 24213162  77886107  64128946 | 24212213  77887043  64129889 | 949  936  943 | cDNA, Ditags, ESTs | |
| **let-7a-1~7f-1~7d** | | Human: 9: 95978060-95981023 [1]  Mouse: 13: 48631381-48633641 [-1]  Rat: 17: 22119767-22121991 [1] | 95968554  48643000  22110767 | 95994023  48630000  22134991 | 25469  13000  24224 | TSS, CpG, 5’CAGE, polyA | |
| **mir-30d~30b** | | Human: 8: 135881945-135886370 [-1]  Mouse: 15: 68168977-68172851 [-1]  Rat: 7: 105815917-105819801 [-1] | 135914350  68195000  105845801 | 135871945  68158977  105805917 | 42405  36023  39884 | TSS, CpG, 5’CAGE, ESTs, polyA | |
| **let-7a-3~7b** | | Human: 22: 44887293-44888312 [1]  Mouse: 15: 85537033-85537833 [1]  Rat: 7: 123700790-123701289 [1] | 44886200  85502033  123665790 | 44889312  85538833  123702289 | 3112  36800  36499 | TSS, CpG, 5’CAGE, polyA, ESTs | |
| **mir-99b~let-7e~mir-125a** | | Human: 19: 56887677-56888404 [1]  Mouse: 17: 17967152-17967843 [1]  Rat: 1: 56486969-56487664 [1] | 56884026  17963852  56483669 | 56888404  17968000  56488064 | 4378  4148  4395 | TSS, CpG, 5’CAGE, ESTs | |
| **mir-29b-2~29c** | | Human: 1: 206041820-206042491 [-1]  Mouse: 1: 196863234-196863828 [1]  Rat: 13: 110967520-110968135 [1] | 206044048  196863023  110967431 | 206041490  196865102  110969284 | 2558  2079  1853 | 5’CAGE, Ditags, polyA, ESTs, cDNA | |
| **mir-183~96~182** | | Human: 7: 129197459-129202090 [-1]  Mouse: 6: 30115918-30119737 [-1]  Rat: 4: 57074891-57075203 [-1] | 129207090  30124737  57080203 | 129186259  30104718  57063691 | 20831  20019  16512 | TSS, CpG, 5’CAGE, polyA | |
| **mir-29b-1~29a** | | Human: 7: 130212046-130212838 [-1]  Mouse: 6: 31012660-31013093 [-1]  Rat: 4: 58107760-58108219 [-1] | 130450838  31063672  58273219 | 130172046  30972660  58067760 | 278792  91012  205459 | TSS, CpG, 5’CAGE, ESTs, cDNA, polyA | |
| **mir-181c~181d** | | Human: 19: 13846513-13846825 [1]  Mouse: 8: 86702615-86702860 [-1]  Rat: 19: 25667384-25667649 [1] | 13837313  86697630  25662384 | 13849063  86709116  25669000 | 11750  11486  6616 | TSS, CpG, 5’CAGE, ESTs | |
| **mir-23a~27a~24-2** | | Human: 19: 13808101-13808473 [-1]  Mouse: 8: 86732417-86732820 [1]  Rat: 19: 25638430-25638818 [-1] | 13811526  86724500  25646500 | 13805101  86733820  25637230 | 6425  9320  9270 | TSS, CpG, 5’CAGE, polyA, Ditags, ESTs, cDNA | |
| **mir-374b~421** | | Human: X: 73354937-73355178 [-1]  Mouse: X: 100768260-100768493 [-1]  Rat: X: 91563416-91563639 [-1] | 73358478  100768793  91563939 | 73354000  100765260  91560416 | 4478  3533  3523 | Ditags, 5’CAGE, ESTs | |
| **mir-34b~34c** | | Human: 11: 110888873-110888956 [1]  Mouse: 9: 50911139-50911750 [-1]  Rat: 8: 54422052-54422653 [-1] | 110886535  50913750  54424653 | 110889820  50910292  54421161 | 3285  3458  3492 | TSS, CpG, ESTs, cDNA, polyA | |
| **hsa-mir-200b~200a~429** | | Human: 1: 1092347-1094330 [1] | 1087121 | 1093585 | 6464 | TSS, CpG, ESTs, 5’CAGE, Ditags | |
| **hsa-mir-200c~141** | | Human: 1: 6943123-6943615 [1] | 6942650 | 6943861 | 1211 | TSS, CpG, ESTs, Ditags, polyA | |
| **let-7i** | | Human: 12: 61283733-61283816 [1]  Mouse: 10: 122422696-122422780 [-1]  Rat: 7: 62755360-62755444 [-1] | 61283533  122422980  62755644 | 61284136  122422376  62755040 | 603  604  604 | TSS, CpG, 5’CAGE, Ditags, ESTs | |
| **mir-124-1** | | Human: 8: 9798308-9798392 [-1]  Mouse: 14: 65209494-65209578 [1]  Rat: 15: 43944536-43944620 [1] | 9801692  65206194  43941000 | 9795008  65212878  43947920 | 6684  6684  6920 | TSS, CpG, 5’CAGE, ESTs, cDNA, polyA | |
| **mir-124-2** | | Human: 8: 65454260-65454368 [1]  Mouse: 3: 17695662-17695770 [1]  Rat: 2: 102699466-102699574 [1] | 65444469  17686662  102690466 | 65458368  17699770  102703574 | 13899  13108  13108 | TSS, CpG, ESTs, cDNA, polyA | |
| **mir-124-3** | | Human: 20: 61280297-61280383 [1]  Mouse: 2: 180628745-180628812 [1]  Rat: 3: 170004571-170004657 [1] | 61278297  180627000  170003000 | 61285383  180630000  170009657 | 7086  3000  6657 | TSS, CpG, 5’CAGE, ESTs, polyA | |
| **mir-129-1** | | Human: 7: 127635161-127635232 [1]  Mouse: 6: 28972619-28972691 [1]  Rat: 4: 55892162-55892233 [1] | 127586875  28931619  55851162 | 127648232  28985691  55905233 | 61357  54072  54071 | TSS, CpG, 5’CAGE, polyA, ESTs | |
| **mir-130a** | | Human: 11: 57165247-57165335 [1]  Mouse: 2: 84581272-84581335 [-1]  Rat: 3: 67949594-67949681 [-1] | 57162247  84584335  67952681 | 57175335  84571272  67950000 | 13088  13063  2681 | TSS, 5’CAGE, ESTs, cDNA | |
| **mir-135a-2** | | Human: 12: 96481721-96481820 [1]  Mouse: 10: 91534831-91534930 [-1]  Rat: 7: 29077659-29077758 [-1] | 96411720  91604900  29090000 | 96483020  91503631  29076459 | 71300  101269  13541 | TSS, ESTs, cDNA, Ditags | |
| **mir-196a-1** | | Human: 17: 44064851-44064920 [-1]  Mouse: 11: 96126478-96126579 [1]  Rat: 10: 84995948-84996027 [1] | 44066920  96124422  84993948 | 44063351  96128028  84997527 | 3569  3606  3579 | TSS, CpG, polyA, ESTs | |
| **mir-203** | | Human: 14: 103653495-103653604 [1]  Mouse: 12: 113369091-113369166 [1]  Rat: 6: 136932089-136932185 [1] | 103652995  113368591  136931589 | 103655104  113370666  136933685 | 2109  2075  2096 | TSS, CpG, 5’CAGE, polyA, ESTs | |
| **mir-210** | | Human: 11: 558089-558198 [-1]  Mouse: 7: 148407283-148407392 [-1]  Rat: 1: 201415656-201415765 [1] | 558598  148407792  201415256 | 555589  148404783  201418265 | 3009  3009  3009 | TSS, CpG, 5’CAGE, cDNA, polyA | |
| **mir-219-1** | | Human: 6: 33283590-33283699 [1]  Mouse: 17: 34161928-34162037 [-1]  Rat: 20: 4967801-4967910 [1] | 33283290  34162337  4967501 | 33284199  34161600  4968200 | 909  737  699 | CpG, Ditags, ESTs | |
| **mir-9-3** | | Human: 15: 87712252-87712341 [1]  Mouse: 7: 86650150-86650239 [1]  Rat: 1: 135254133-135254222 [1] | 87700000  86643950  135247933 | 87730000  86678239  135282222 | 30000  34289  34289 | TSS, CpG, 5’CAGE, cDNA, ESTs, polyA | |
| **mir-92b** | | Human: 1: 153431592-153431687 [1]  Mouse: 3: 89031038-89031120 [-1]  Rat: 2: 181395426-181395508 [-1] | 153431000  89031320  181395708 | 153431987  89030738  181395126 | 987  582  582 | TSS, CpG, Ditags, 5’CAGE, ESTs | |
| **mir-34a** | | Human: 1: 9134314-9134423 [-1]  Mouse: 4: 149442563-149442664 [1]  Rat: 5: 167187897-167187998 [1] | 9154423  149422563  167167897 | 9131014  149445964  167191298 | 23409  23401  23401 | polyA, ESTs | |
| **mir-223** | | Human: X: 65155437-65155546 [1]  Mouse: X: 93438156-93438265 [1]  Rat: X: 83869053-83869162 [1] | 65152028  93435281  83866053 | 65156989  93438975  83869965 | 4961  3694  3912 | ESTs, cDNA, 5’CAGE, polyA | |
| **hsa-mir-21** | | Human:17: 55273409-55273480 [1] | 55271009 | 55274397 | 3388 | ESTs, cDNA, 5’CAGE, Ditags, polyA | |
| **hsa-mir-125b-1** | | Human:11: 121475675-121475762 [-1] | 121478084 | 121465024 | 13060 | ESTs, cDNA, 5’CAGE, polyA | |
| **hsa-mir-9-2** | | Human: 5: 87998427-87998513 [-1] | 88004913 | 87996020 | 8893 | TSS, CpG, 5’CAGE, cDNA, ESTs, polyA | |
| **mir-219-2** | | Human: 9: 130194718-130194814 [-1]  Mouse: 2: 29701151-29701247 [-1] | 130195207  29702004 | 130194122  29700562 | 1085  1442 | TSS, CpG, 5’CAGE, cDNA, ESTs, Ditags, polyA | |
| **mir-101a** | | Human: 1: 65296705-65296779 [-1]  Mouse: 4: 101019550-101019632 [-1]  Rat: 5: 121990585-121990659 [-1] | 65305879  101028814  122000659 | 65296205  101019136  121990085 | 9674  9678  10574 | TSS, CpG, 5’CAGE, ESTs, cDNA, | |
| **mmu-mir-551b** | | Mouse: 3: 29315745-29315842 [1] | 28981483 | 29415524 | 434041 | TSS, CpG, 5’CAGE, cDNA, ESTs, Ditags, polyA | |
| **Group II pri-miRNAs** | | | | | | | |
| **mir-15a~16-1** | | Human: 13: 49521110-49521338 [-1]  Mouse: 14: 62250717-62250947 [-1] | 49553338  62300947 | 49516110  62245717 | 37228  55230 | TSS, CpG, 5’CAGE, Ditags, polyA, ESTs, cDNA | |
| **mir-193b~365-1** | | Human: 16: 14305325-14310729 [1]  Mouse: 16: 13449616-13454019 [1] | 14303625  13447916 | 14327729  13471019 | 24104  23103 | TSS, CpG, ESTs, 5’CAGE, polyA | |
| **mir-148a** | | Human: 7: 25956064-25956131 [-1]  Mouse: 6: 51219811-51219909 [-1] | 25957131  51220909 | 25946064  51209811 | 11067  11098 | TSS, CpG, 5’CAGE, polyA, ESTs | |
| **mir-155** | | Human: 21: 25868163-25868227 [1]  Mouse: 16: 84714385-84714449 [1] | 25856363  84702585 | 25870327  84716549 | 13964  13964 | TSS, CpG, 5’CAGE, ESTs, cDNA, | |
| **Group III pri-miRNAs** | | | | | | | |
| **hsa-mir-374a~545** | | X: 73423664-73423917 [-1] | 73430117 | 73416664 | 13453 | TSS, 5’CAGE, ESTs, cDNA, polyA | |
| **hsa-mir-548a-1** | | 6: 18679994-18680090 [1] | 18630994 | 18831090 | 200096 | TSS, cDNA, polyA | |
| **hsa-mir-550-2** | | 7: 32739118-32739214 [1] | 32735018 | 32761214 | 26196 | TSS, CpG, cDNA, ESTs, polyA | |
| **mmu-mir-344-1** | | 7: 69022656-69022750 [-1] | 69070245 | 68851316 | 218929 | Ditags, cDNA | |
| **Group IV pri-miRNAs** | | | | | | | |
| **hsa-mir-297** | 4: 112001187-112001252 [-1] | | 112305452 | 111998187 | 307265 | | Ditags |
| **hsa-mir-557** | 1: 166611386-166611483 [1] | | 166608486 | 166612083 | 3597 | | Ditags |
| **hsa-mir-572** | 4: 10979549-10979643 [1] | | 10979324 | 10984443 | 5119 | | TSS, CpG, 5’CAGE, ESTs, polyA, Ditags |
| **hsa-mir-583** | 5: 95440598-95440672 [1] | | 95438309 | 95440829 | 2520 | | Ditags |
| **hsa-mir-588** | 6: 126847470-126847552 [1] | | 126808057 | 126951400 | 143343 | | Ditags |
| **hsa-mir-596** | 8: 1752804-1752880 [1] | | 1752104 | 1754880 | 2776 | | TSS, CpG, ESTs, polyA |
| **hsa-mir-602** | 9: 139852692-139852789 [1] | | 139852178 | 139853450 | 1272 | | Ditags, polyA |
| **hsa-mir-612** | 11: 64968505-64968604 [1] | | 64967403 | 64968986 | 1583 | | Ditags |
| **hsa-mir-614** | 12: 12960030-12960119 [1] | | 12958330 | 12962219 | 3889 | | polyA, cDNA |
| **hsa-mir-633** | 17: 58375308-58375405 [1] | | 58350238 | 58553505 | 203267 | | Ditags |
| **hsa-mir-648** | 22: 16843634-16843727 [-1] | | 16876592 | 16824114 | 52478 | | Ditags, polyA |
| **hsa-mir-663** | 20: 26136822-26136914 [-1] | | 26137914 | 26115622 | 22292 | | TSS, CpG, 5’CAGE, cDNA, polyA |
| **mmu-mir-327** | 14: 45567118-45567186 [-1] | | 45572986 | 45566894 | 6092 | | Ditags |
| **mmu-mir-568** | 16: 43640768-43640850 [1] | | 43639978 | 43642390 | 2412 | | 5’CAGE, Ditags, cDNA, ESTs, polyA |
| **mmu-mir-715** | 17: 39981081-39981190 [1] | | 39979958 | 39982700 | 2742 | | TSS, 5’CAGE, ESTs, cDNA, Ditags |
